# The prognostic value of quality of life in atrial fibrillation on patient value

**DOI:** 10.1186/s12955-023-02112-2

**Published:** 2023-04-05

**Authors:** Luc J.H.J. Theunissen, Jeroen A.A. van de Pol, Gijs J. van Steenbergen, Henricus-Paul Cremers, Dennis van Veghel, Pepijn H. van der Voort, Peter E. Polak, Sylvie F.A.M.S. de Jong, Jaap Seelig, Geert Smits, Hareld M.C. Kemps, Lukas R.C. Dekker

**Affiliations:** 1 Máxima Medical Center, Veldhoven, The Netherlands; 2 Netherlands Heart Network, Michelangelolaan 2, Eindhoven, 5623 EJ The Netherlands; 3 Catharina hospital Eindhoven, Eindhoven, The Netherlands; 4 Anna hospital, Geldrop, The Netherlands; 5 Elkerliek hospital, Helmond, The Netherlands; 6 Rijnstate, Arnhem, The Netherlands; 7 Cardiovascular research institute, Maastricht University, Maastricht, The Netherlands; 8 GP Organization POZOB, Veldhoven, The Netherlands; 9 Department of Industrial Design, Eindhoven University of Technology (TUe), Eindhoven, The Netherlands; 10 Department of Electrical Engineering (SPS group), Eindhoven University of Technology (TUe), Eindhoven, The Netherlands

**Keywords:** Quality of life, Atrial fibrillation, MACE, Patient-relevant outcomes, Patient value

## Abstract

**Background:**

In this study, the prognostic value of AF-related quality of life (AFEQT) at baseline on Major Adverse Cardiovascular Events (MACE) and improvement of perceived symptoms (EHRA) was assessed. Furthermore, the relationship between QoL and AF-related hospitalizations was assessed.

**Methods:**

A cohort of AF-patients diagnosed between November 2014 and October 2019 in four hospitals embedded within the Netherlands Heart Network were prospectively followed for 12 months. MACE was defined as stroke, myocardial infarction, heart failure and/or mortality. Subsequently, MACE, EHRA score improvement and AF-related hospitalizations between baseline and 12 months of follow-up were recorded.

**Results:**

In total, 970 AF-patients were available for analysis. In analyses with patients with complete information on the confounder subset 36/687 (5.2%) AF-patients developed MACE, 190/432 (44.0%) improved in EHRA score and 189/510(37.1%) were hospitalized during 12 months of follow-up. Patients with a low AFEQT score at baseline more often developed MACE (OR(95%CI): 2.42(1.16–5.06)), more often improved in EHRA score (OR(95%CI): 4.55(2.45–8.44) and were more often hospitalized (OR(95%CI): 4.04(2.22–7.01)) during 12 months post diagnosis, compared to patients with a high AFEQT score at baseline.

**Conclusions:**

AF-patients with a lower quality of life at diagnosis more often develop MACE, more often improve on their symptoms and also were more often hospitalized, compared to AF-patients with a higher quality of life. This study highlights that the integration of patient-reported outcomes, such as quality of life, has the potential to be used as a prognostic indicator of the expected disease course for AF.

**Supplementary Information:**

The online version contains supplementary material available at 10.1186/s12955-023-02112-2.

## Introduction

Atrial fibrillation (AF) is the most common cardiac arrhythmia in adults [1, 2]. As a result of the aging population and early screening initiatives, the incidence and prevalence of AF are expected to increase in future decades with 17.9 million Europeans suffering from AF by 2060 [1, 3]. The increased proportion of older adults who suffer from AF will have several impactful consequences for public health, including higher disease burden, health service utilization and health care costs [1, 4]. Therefore, there is an urgent need for new strategies to improve patient-relevant outcomes and decrease healthcare costs.

AF often leads to the occurrence of various concomitant cardiovascular disorders with a prominent effect on the patients’ disease burden such as major adverse cardiovascular events (MACE), a composite of myocardial infarctions (MI), stroke, heart failure and/or mortality. In AF, the occurrence of MACE is perceived as the most relevant outcome in secondary prevention [5]. In addition to MACE, AF also commonly features symptoms that influence the patients’ capabilities to undertake daily activities. The extent of the patients’ limitations and symptoms are routinely assessed in clinical practice using the European Heart Rhythm Association score of atrial fibrillation (EHRA) classification system [6]. Palpitations, exercise intolerance, dizziness, dyspnea at rest and chest discomfort and/or tightness are commonly experienced symptoms by AF-patients that have been shown to negatively affect the quality of life (QoL) of patients [7, 8].

As a result of concomitant cardiovascular disorders, bleedings and underlying non-cardiovascular conditions AF patients are often hospitalized [9, 10]. In general, approximately 30% of AF patients are hospitalized at least once per year, while 10% are hospitalized twice or more per year [10]. The largest part of healthcare costs for AF-patients can be accounted for by (the length of) hospitalizations and in-hospital procedures as a result of comorbidities [11–13]. Even though a relationship has been established between QoL and adverse outcomes in AF, limited information is available on the relationship between QoL at diagnosis and patient-relevant outcomes in AF during the disease trajectory. Being able to predict the occurrence of patient-relevant outcomes and hospitalizations should prove incredibly valuable for individual tailoring of AF treatment during the disease course. Focusing on patient-relevant outcomes and critically examining healthcare costs and utilization early in the disease course for new AF patients may enable medical specialists to focus on improving patient value.

Patient value is defined as patient-relevant outcomes divided by the costs of healthcare delivery and is the core philosophy of value-based healthcare (VBHC) [14]. VBHC was originally introduced by Porter and Teisberg as a strategy to improve quality in healthcare, reduce variation in outcomes that matter most to patients, raise awareness for the emerging cost crisis in healthcare and to put patient value central in the delivery of care [14]. By identifying potential predictors for future patient-relevant outcomes and healthcare costs early in the disease trajectory potential interventional strategies can be employed to help reduce the burden of AF patients and potentially reduce healthcare costs. An emerging topic to estimate AF disease trajectories is the use of patient-derived outcome measures such as QoL [15]. QoL at diagnosis could potentially be an early indicator of future patient-relevant outcomes and healthcare costs [15].

Therefore, the aim of this study was to assess the association between QoL and both patient-relevant outcomes and hospitalizations as a proxy for healthcare costs in AF patients. To this end, we assessed the association between QoL as measured by the Atrial Fibrillation Effect on QualiTy-of-life (AFEQT) questionnaire, and EHRA score improvement, the occurrence of MACE and hospitalizations in Dutch AF-patients.

## Methods

### Study design

This prospective cohort study was performed using information from newly diagnosed AF patients between November 2014 and October 2019 in the Southeast of the Netherlands with a catchment population of approximately 800.000 inhabitants. Within this region, four non-university hospitals and approximately 350 general practitioner (GP) practices embedded within the Netherlands Heart Network (NHN) work together to improve patient-relevant outcomes and lower healthcare costs for cardiac patients across the whole healthcare chain in collaboration with all relevant healthcare providers in primary, secondary and tertiary care. Within the NHN, regional and transmural care is evaluated based on patient value according to the VBHC philosophy [16].

### Procedure and population

Within the NHN, the collaborating hospitals and GP practices have developed and implemented a regional standard of care protocol aimed at guiding physicians in the management of AF patients [16]. As part of this care pathway, AF patients are educated about available treatment options and the importance of treatment compliance by specialized and trained AF-nurses. In addition, the AF-nurse registers information on patient characteristics, the patients’ general health status, and AF-related complaints to aid the shared decision-making process and patient counselling by medical specialists. Information includes, among others, patient demographics, patient characteristics, patient vitals, AF-related risk stratification scores, onset of symptoms, and an AF-related Quality of Life (QoL) questionnaire. AF-patients included in the study were followed-up after 12 months (T1) to record patient characteristics, the occurrence of patient-relevant outcomes and to evaluate the initiated treatment.

During the initial visit, AF-nurses also assessed study eligibility, provided information on the study, registered patient information and obtained written informed consent. Eligibility criteria for inclusion were: age ≥ 18 years, a new or recent diagnosis with non-valvular atrial fibrillation, competence to read and agree on the informed consent, and provision of written informed consent. Patients with impaired cognition and the inability to understand Dutch were excluded.

### Ethical approval

The protocol of the AF-NET study was submitted for approval to the Medical research Ethics Committee United (MEC-U) in the Netherlands (reference number: 14.083). The MEC-U confirmed that the Medical Research Involving Human Subjects Act does not apply to the AF-NET study and, therefore, an official approval of this study by the MEC-U is not required.

### Assessment of AF-related Effect on QualiTy of life (AFEQT) and baseline measurement

Baseline characteristics were measured during the routine visit (T0) at an AF-outpatient clinic visit by dedicated AF-nurses. Among these baseline characteristics was the exposure of interest, namely the patient-reported QoL as measured through the AFEQT questionnaire [17]. The AFEQT is a validated and reliable 20-item questionnaire developed to quantify QoL in AF-patients across 4 conceptual domains (Symptoms, Daily Activities, Treatment Concerns and Treatment Satisfaction) using a 7-point Likert response scale [17]. The AFEQT questionnaire was provided to patients by AF-nurses at the initial visit to the AF-outpatient clinic. Completed questionnaires were returned to the AF-nurses and answers were registered by data registration employees. The overall score on the AFEQT questionnaire is calculated using answers from the first three subdomains and ranges from 0 (severe impairment/low QoL) to 100 (no limitation/high QoL), according to the guidelines of the AFEQT scoring manual [18]. The treatment satisfaction domain was not considered in this study as AFEQT questionnaires were collected within a short period after initial diagnosis. Resultingly, patients may not yet have experienced the full effects of treatment, which may affect the accuracy of responses regarding this domain.In this study, patients were categorized into quartiles, using the upper quartile (high QoL) as the reference, based on their final AFEQT scores observed in this study (AFEQT score 0: >90.74; 1: >75.9 to ≤ 90.74; 2: >57.41 to ≤ 75.93; 3: ≤57.41).

Additionally, various other baseline characteristics were recorded, including: age, gender, a composite of Congestive heart failure or left ventricular dysfunction Hypertension, Age ≥ 75 (doubled), Diabetes, Stroke, (doubled)-Vascular disease, Age 65–74, Sex category (female) (CHA_2_DS_2_-VASc) score [19], a composite of Hypertension, Abnormal renal/liver function, Stroke, Bleeding history or predisposition, Labile INR, Elderly, Drugs/alcohol concomitantly (HAS-BLED) score [20], Body Mass Index (BMI), diabetes mellitus (DM), hypertension, obstructive sleep apnea syndrome (OSAS), prior heart failure, malignancy, chronic lung disease, and location of AF diagnosis (General practitioner/Hospital). Background variables were selected for use in this study based on availability and inclusion as cardiovascular risk factors in guidelines from the European Society of Cardiology (ESC)[1].

### Outcome measures

The extent of AF-related symptoms was measured using the EHRA classification score of atrial fibrillation [6]. The EHRA score was used as a specific, yet simple, quantification of the functional consequences of AF, as reported by the clinician. The EHRA score is a 4-point scale which ranges from low symptom severity (EHRA I: no symptoms; normal daily activity not affected) to high symptom severity (EHRA IV: disabling symptoms; normal daily activity discontinued) [6]. EHRA improvement was determined by comparing the EHRA score at 12 months of follow-up (T1) with the EHRA score at time of diagnosis (T0). Any full point improvement in EHRA score was perceived as clinically relevant, hence the use of the unmodified EHRA score during this study. MACE was defined as the composite of any MI, stroke, heart failure and mortality between baseline and 12 months of follow-up. AF-nurses assessed whether patients were hospitalized between baseline and 12 months of follow-up by checking their hospital record during routine follow-up. If patients had any AF-related hospital visit during 12 months of follow-up, a hospitalization was recorded.

### Statistical analysis

Patient characteristics at baseline were described using means, standard deviations (SD) and proportions (%). Minimally and multivariable-adjusted logistic regression analyses (Odds Ratios (ORs) and 95% confidence intervals (CIs)) were performed to assess the association between AFEQT score at baseline (T0) and the occurrence of MACE, the improvement of EHRA score, and AF-related hospitalizations between baseline (T0) and 12 months of follow-up (T1). Minimally-adjusted analyses were adjusted for categorized age and gender. In addition to categorized age and gender, in multivariable-adjusted analyses type of AF, CHA_2_DS_2_-VASc score, HAS-BLED score were included in all multivariable-adjusted models as a priori confounders. Potential other confounders (i.e. Overweight (BMI ≥ 25 kg/m^2^), DM, hypertension, OSAS, heart failure, malignancy, chronic lung disease and location of AF diagnosis) were added to the multivariable-adjusted model using backwards elimination (p < 0.10). Based on this procedure DM was included in statistical models related to EHRA improvement. No additional potential confounders were included in statistical models related to MACE and hospitalizations. In sensitivity analyses in which the complete confounder subset was included, results were similar to the main analyses (Supplementary material S1.1-S1.3). In addition, a sensitivity analysis was performed to assess potential floor effects regarding the relationship between AFEQT and EHRA score improvement (Supplementary material S1.4). No multicollinearity was observed in tests between the CHA_2_DS_2_-VASc and HAS-BLED scores. As a result, all models included both CHA_2_DS_2_-VASc and HAS-BLED. Missing values were handled using listwise deletion on a per analysis basis using the final multivariable-adjusted model. Due to the limited number of cases who developed MACE during 12 months of follow-up (n = 36) statistical analyses using quartiles were underpowered for analysis (< 5 participants in one quartile). As a result, AFEQT categories were categorized based on the median overall AFEQT score to gain insights in the association between AF-related quality of life and the development of MACE. All analyses were performed using International Business Machines corporation Statistical Package for the Social Sciences (IBM SPSS) (IBM SPSS Statistics for Windows, version 26.0, IBM Corp., Armonk, NY). P-values < 0.05 were considered statistically significant.

## Results

In total, 970 AF-patients were available for analysis based on the availability of an AFEQT questionnaire at baseline. Baseline characteristics of AF-patients, categorized into quartiles based on the AFEQT score ranging from low QoL (Q1) to high QoL (Q4), are presented in Table 1. Based on the calculated AFEQT quartiles, 239 AF-patients were in Q1 (AFEQT-score (4.63 to ≤ 57.41)), 238 in Q2 (> 57.41 to ≤ 75.93), 248 in Q3 (> 75.93 to ≤ 90.74), and 245 in Q4 (> 90.74 to 100). Compared to patients with a high AFEQT score (Q4), patients with a lower AFEQT score (Q1) were more often female (Q1; 54.8% vs. Q4; 28.6%), more often had a CHA_2_DS_2_VASc score of 2+ (Q1; 77.4% vs. Q4; 67.5%), more often had a HAS-BLED score of 2+ (Q1; 49.0% vs. Q4; 40.9%), had a higher prevalence of DM (Q1; 15.5% vs. Q4; 12.2%), were more often overweight or obese (Q1; 73.9% vs. Q4; 65.7%), more often had hypertension (Q1; 59.4% vs. Q4; 50.2%), more often had heart failure at baseline (Q1; 7.5% vs. Q4; 1.6%), more often had chronic lung disease (Q1; 12.7% vs. Q4; 7.0%) and had a lower EHRA score at baseline (mean (SD); Q1; 2.29 (0.90) vs. Q4; 1.42 (0.62)). Patients in AFEQT score quartiles Q2 and Q3 less often suffered from persistent AF, compared to patients in AFEQT score quartiles Q1 and Q4 (Q2; 25.4% and Q3; 25.9% vs. Q1; 37.1% and Q4; 34.0%, respectively).

**Table 1:** Baseline characteristics of AF-patients categorized into quartiles based on the AFEQT score at baseline

	AFEQT score at baseline				
	*First quartile (Q1)* *(4.63 to ≤ 57.41)*	*Second quartile (Q2)(> 57.41 to ≤ 75.93)*	*Third quartile (Q3)* *(> 75.93 to ≤ 90.74)*	*Fourth quartile (Q4)(> 90.74 to 100)*	*p-value*
	n (%)	n (%)	n (%)	n (%)	
Total*	239 (24.6%)	238 (24.5%)	248 (25.6%)	245 (25.3%)	-
Gender					
Man	108 (45.2%)	136 (57.1%)	141 (56.9%)	175 (71.4%)	
Woman	131 (54.8%)	102 (42.9%)	107 (43.1%)	70 (28.6%)	< 0.001
Age					
mean (SD)	70.0 (10.4)	69.7 (9.7)	69.1 (9.6)	69.1 (9.1)	0.667
Type of AF					
Paroxysmal	132 (62.9%)	156 (74.6%)	163 (73.1%)	140 (66.0%)	
Persistent	78 (37.1%)	53 (25.4%)	60 (26.9%)	72 (34.0%)	0.024
CHA_2_DS_2_-VASc score (T0)					
0–1	54 (22.6%)	52 (22.3%)	66 (26.8%)	79 (32.5%)	
2+	185 (77.4%)	181 (77.7%)	180 (73.2%)	164 (67.5%)	0.037
HAS-BLED (T0)					
0–1	104 (51.0%)	120 (62.8%)	120 (60.0%)	117 (59.1%)	
2+	100 (49.0%)	71 (37.2%)	80 (40.0%)	81 (40.9%)	0.095
OSAS					
No	225 (94.1%)	229 (96.2%)	232 (93.9%)	233 (95.1%)	
Yes	14 (5.9%)	9 (3.8%)	15 (6.1%)	12 (4.9%)	0.656
Diabetes mellitus					
No	202 (84.5%)	203 (85.3%)	217 (87.5%)	215 (87.8%)	
Yes	37 (15.5%)	35 (14.7%)	31 (12.5%)	30 (12.2%)	0.663
BMI^a^					
< 25	55 (26.1%)	68 (33.7%)	66 (32.8%)	70 (34.3%)	
≥ 25	156 (73.9%)	134 (66.3%)	135 (67.2%)	134 (65.7%)	0.239
Hypertension					
No	97 (40.6%)	103 (43.3%)	105 (42.3%)	122 (49.8%)	
Yes	142 (59.4%)	135 (56.7%)	143 (57.7%)	123 (50.2%)	0.187
Heart failure					
No	221 (92.5%)	229 (96.2%)	243 (98.0%)	240 (98.4%)	
Yes	18 (7.5%)	9 (3.8%)	5 (2.0%)	4 (1.6%)	0.002
Malignancy					
No	209 (87.4%)	210 (88.6%)	222 (89.5%)	214 (87.7%)	
Yes	30 (12.6%)	27 (11.4%)	26 (10.5%)	30 (12.3%)	0.890
Chronic lung disease					
No	207 (87.3%)	212 (89.1%)	229 (26.2%)	227 (93.0%)	
Yes	30 (12.7%)	26 (10.9%)	19 (7.7%)	17 (7.0%)	0.107
EHRA at baseline					
Mean (SD)	2.29 (0.90)	1.85 (0.83)	1.69 (0.80)	1.42 (0.65)	< 0.001
Location of diagnosis					
General practitioner	79 (33.6%)	62 (26.3%)	79 (32.1%)	78 (31.8%)	
Hospital	156 (66.4%)	174 (73.7%)	167 (67.9%)	167 (68.2%)	0.328

Abbreviations: SD: Standard Deviation, T0: baseline, OSAS: Obstructive Sleep Apnea Syndrome, BMI: Body Mass Index

^*^ Numbers may not add up to total due to missing values for individual parameters

### Occurrence of MACE during 12 months of follow-up

Based on complete information for confounders and the development of MACE 687 AF-patients were available for analysis. In total, 36 (5.2%) of all patients developed MACE during follow-up (Table 2). Due to the low frequency of occurrence of MACE and the resulting limited power, AFEQT scores were assessed using the median score. In multivariable-adjusted analyses, AFEQT scores below the median (75.93) at baseline were associated with a statistically significantly increased odds of developing MACE during 12 months of follow-up, when compared to patients with AFEQT scores above the median at baseline (OR (95% CI); 2.42 (1.16–5.06)). Results for minimally-adjusted analyses were similar in direction to multivariable-adjusted analyses, albeit mildly attenuated.

**Table 2:** Overall associations between QoL at baseline (AFEQT) and the occurrence of MACE after 12 months of follow-up (T1)

	Total study population		MACE^1^ (T1-T0)		
AFEQT score (T0)	n (%)	n (%)	OR_minimally−adjusted_ (95% CI)	OR_mv−adjusted_ (95% CI)	*p-value*
Below median (4.63 to ≤ 75.93)	339 (49.3%)	25 (7.4%)	2.55 (1.23–5.29)	2.42 (1.16–5.06)	0.018
Above median (> 75.93 to 100)	348 (50.7%)	11 (3.2%)	1 (ref.)	1 (ref.)	

Minimally-adjusted models were adjusted for categorized age (< 65; ≥65) and gender

Multivariable-adjusted models were additionally adjusted for HAS_BLED (0–1; ≥2), CHA2DS2-VASc (0–1; ≥2), type of AF (paroxysmal/persistent)

^1^ MACE was defined as the composite of any MI/stroke (n=14), heart failure (n=20) and mortality (n=8).

### EHRA improvement after 12 months

Based on complete information for confounders and EHRA improvement 432 AF-patients were available for analysis. In total, 190 (44.0%) AF-patients improved in EHRA-score within 12 months. A weak correlation was observed between EHRA and AFEQT at baseline (r=-0.359). Results from multivariable-adjusted analyses on the association between AFEQT score and the improvement in EHRA score after 12 months of follow-up are presented in Table 3. The improvement in EHRA score was statistically significant across all quartiles of AFEQT score and associations became stronger across decreasing AFEQT scores, when compared to patients in the highest AFEQT quartile (Q1 vs. Q4: OR (95% CI); 4.55 (2.45–8.44)). Results for minimally-adjusted analyses were similar in strength and direction, when compared to multivariable-adjusted analyses. In sensitivity analyses, floor effects were tested. In these analyses, in which AF-patients with EHRA 1 were excluded, associations attenuated and became not statistically significant (supplementary material S1.4). However, similar to primary analyses, the association became stronger in patients with an increasingly lower AFEQT score at baseline with OR’s ranging from 1.38 to 1.69 (p-value: 0.439 − 0.232). In part, the attenuation of associations is likely attributable to the presence of floor effects. However, in this analysis, the statistical power decreased both due to the lower number of AF-patients available for analysis (nsensitivity = 283 vs. nmain = 432), and due to the disproportionate reduction of patients in the reference category (Q4), which may explain the lack of statistical significance.

**Table 3:** Overall associations between QoL at baseline (AFEQT) and the improvement in symptom scores (EHRA improvement) after 12 months (T1)

	Total study population		EHRA improvement (T1-T0)		
AFEQT score (T0)	n (%)	n (%)	OR_minimally−adjusted_ (95% CI)	OR_mv−adjusted_ (95% CI)	*p-value*
First quartile (4.63 to ≤ 57.41)	121 (28.0%)	69 (57.0%)	4.41 (2.41–8.08)	4.55 (2.45–8.44)	< 0.001
Second quartile (> 57.41 to ≤ 75.93)	97 (22.5%)	50 (51.5%)	3.53 (1.88–6.62)	3.42 (1.80–6.53)	< 0.001
Third quartile (> 75.93 to ≤ 90.74)	116 (26.9%)	49 (42.2%)	2.44 (1.33–4.48)	2.34 (1.26–4.34)	0.007
Fourth quartile (> 90.74 to 100)	98 (22.7%)	22 (22.4%)	1 (ref.)	1 (ref.)	

Minimally-adjusted models were adjusted for categorized age (< 65; ≥65) and gender

Multivariable-adjusted models were additionally adjusted for HAS_BLED (0–1; ≥2), CHA_2_DS_2_VASc (0–1; ≥2), type of AF (paroxysmal/persistent), Diabetes Mellitus

### Occurrence of AF-related hospitalizations during 12 months of follow-up

Based on complete information for confounders and AF-related hospitalizations 510 AF-patients were available for analysis. In total, 189 (37.1%) of all patients were hospitalized at least once during 12 months of follow-up (Table 4). In multivariable-adjusted analyses, a lower AFEQT score at baseline was statistically significantly associated with an increased risk of hospitalizations in the first quartile (OR (95% CI); 4.04 (2.33–7.01)), when compared to patients with a high AFEQT score at baseline. No statistically significant association was observed between AFEQT and hospitalizations between the second, third and fourth AFEQT quartiles. Results for minimally-adjusted analyses were similar in strength and direction, when compared to multivariable-adjusted analyses.

**Table 4:** Overall associations between QoL at baseline (AFEQT) and AF-related hospitalizations during 12 months of follow-up (T1)

	Total study population		Hospitalizations (T1-T0)		
AFEQT score (T0)	n (%)	n (%)	OR_minimally−adjusted_ (95% CI)	OR_mv−adjusted_ (95% CI)	*p-value*
First quartile (4.63 to ≤ 57.41)	141 (27.6%)	81 (57.4%)	3.86 (2.26–6.59)	4.04 (2.33–7.01)	< 0.001
Second quartile (> 57.41 to ≤ 75.93)	114 (22.4%)	39 (34.2%)	1.50 (0.85–2.64)	1.77 (0.98–3.18)	0.057
Third quartile (> 75.93 to ≤ 90.74)	133 (26.1%)	38 (28.6%)	1.15 (0.66–2.02)	1.27 (0.71–2.25)	0.417
Fourth quartile (> 90.74 to 100)	122 (22.9%)	31 (25.4%)	1 (ref.)	1 (ref.)	

Minimally-adjusted models were adjusted for categorized age (< 65; ≥65) and gender

Multivariable-adjusted models were additionally adjusted for HAS_BLED (0–1; ≥2), CHA_2_DS_2_-VASc (0–1; ≥2), type of AF (paroxysmal/persistent)

## Discussion

The present study aimed to assess the association between QoL at baseline and the occurrence of MACE, EHRA improvement and hospitalizations during 12 months of follow-up. In short, patients with a QoL below the median more often developed MACE, compared to patients with a higher QoL. In addition, patients with a low QoL at baseline more often improved on their AF-related symptoms (EHRA score) during follow-up, compared to patients with a higher QoL. Lastly, patients with a lower QoL were more likely to be hospitalized in the first 12 months after diagnosis, compared to patients with a higher QoL.

Patient-reported outcomes (PROs), such as QoL, are increasingly employed to assess the effects of a health condition and its management on the experienced disease burden and treatment satisfaction of patients and caregivers. Naturally, most studies have primarily focused on the impact of AF on the patients’ QoL [21, 22]. However, aside from evaluating the effects of the experienced disease and QoL, PROs may also hold clinical relevance for predicting future disease trajectories in routine care. QoL is a simple and easily attainable PRO that may be promising for use in risk stratification in everyday clinical practice. To our knowledge, no studies have been published regarding the association between QoL at diagnosis and the subsequent development of MACE during follow-up in a broad spectrum of AF-patients. A previous study by Pedersen et al., which examined cardiac patients after percutaneous coronary intervention (PCI), reported that a poor QoL after PCI was related to the occurrence of MACE within 6 months after percutaneous coronary intervention, but not late MACE [23]. In our study we did not make a distinction in the timing of MACE, which may warrant further investigation in future research as we strictly assessed the occurrence of MACE within 1 year of follow-up.

In addition to MACE, the improvement in EHRA score during the year post-diagnosis was also associated with QoL at diagnosis. Patients with a lower quality of life at diagnosis also more often had a lower EHRA score at diagnosis. Which might indicate that these patients had more opportunity to improve. However, we also observed a weak correlation between AFEQT and EHRA, which indicates that the patients’ perceived health burden is not always in line with the perceived burden as assessed by the doctor. As such, QoL as measured through a dedicated and specialized questionnaire for AF, such as the AFEQT questionnaire, may provide a valuable patient reported outcome to assess the potential for improvement in the patients’ perceived health burden, in conjunction with the EHRA classification as reported by the clinician.

Previous studies have highlighted that there may be a discordance in what patients perceive and what clinicians can detect regarding AF-symptoms [24]. For instance, physicians may underestimate or have difficulty in discriminating mild, low-level, symptoms [24, 25]. As treatment decisions are generally made based on the presence of symptoms to target improvement of AF symptoms in tandem with the expected benefits and risks for the patient, physicians could benefit from more sources of information for deciding on a course of action [24, 26]. Focus groups within the RATE-AF trial have indicated that improvement of QoL, ahead of mortality and hospitalizations, is paramount for AF patients, while patients perceive that healthcare professionals tend to steer on factors which are important to them [27]. Therefore, PROs could help with shifting the focus from symptoms and treatment options to a more patient-centered perspective in clinical care and could contribute in shared-decision making about how to treat AF. To aid this process, disease course prediction by defining traditional risk groups or by artificial intelligence featuring both traditional patient characteristics and symptoms, as well as PROs, may help making well-informed decisions on the preferred treatment regimens, identify areas of improvement and avoiding treatment for patients who are unlikely to benefit from them [28]. This study highlights that patient-reported quality of life may be valuable in cardiac care. More research is needed to develop and validate models which focus on predicting the disease course of patients with atrial fibrillation using input from both patients and clinicians.

Direct healthcare costs for AF are primarily driven by hospitalizations, accounting for 50–70% of total costs [29]. Moreover, these costs are expected to increase in the future due to the ageing population. Therefore, identifying patient groups who have an increased likelihood of becoming hospitalized becomes more important for individualizing treatment [30]. In line with findings from this study, Schron et al. reported that patients’ QoL was a predictor for hospitalization [15]. Notably, Schron et al. employed both the more general Short Form 36 Health Survey Questionnaire (SF-36) and a cardiac-specific QoL (Quality of Life Index-Cardiac Version; QLI-CV) measure [15]. Interestingly, the more general SF-36 summary score led to statistically significant prediction of hospitalizations, while cardiac-specific QoL did not reach statistical significance. In our study, in which we used an AF-specific questionnaire focused on Health Related QoL, QoL was statistically significantly associated with hospitalizations after 1 year, when comparing low vs. high QoL at diagnosis. There were, however, some differences regarding the confounder subsets used in the models and covered domains between the cardiac questionnaires which could explain these differences. Based on these observations QoL may potentially be used as a predictor for hospitalizations and, resultingly, AF-related healthcare costs. A reduced QoL at diagnosis may therefore be used as an indicator for additional surveillance to change treatment regimens before hospitalization occurs.

Overall, from the VBHC perspective, PROs such as QoL as measured by the AFEQT questionnaire could provide valuable opportunities to improve patient value on multiple levels by reducing the occurrence of MACE, facilitating EHRA symptom improvement and reducing AF-related healthcare costs. In addition, the routine implementation of PROMs such as the AFEQT score will empower physicians to treat more than symptoms, but also allow them to focus on patient-perceived improvements and reduction of AF disease burden. For instance, PROs in routine care could aid both clinicians and patients during patient consultations in setting realistic expectations and may aid in the process of shared decision making for treatment options, while taking account the anticipated patient-relevant outcomes and symptom improvements that fit the reported health status of the patient. Moreover, patients’ will benefit from accurately reporting their perceived health status and, in turn, directly impacting their treatment options and outcomes. Furthermore, PROMs may enable machine learning-based initiatives to further refine models and assist in clinical decision making [31]. In this way, patients can attain the best outcomes for their specific medical circumstances. From a managerial perspective, the integration of more patient-centered care allows for a reduction in treatment costs (e.g. reduction of hospitalizations), evaluating performance (e.g. patient-improvement) and improving treatment satisfaction (e.g. shared-decision making) [32]. The integration of PROMs and personalized medicine may, therefore, prove a fruitful avenue for the evaluation of health data, performance assessment, but also for exploring new value-based initiatives [33].

This study was subject to some limitations. Firstly, information on the rate or rhythm control strategy was unavailable. Therefore, we could not control for these variables in our analyses. Secondly, all outcomes were assessed after a follow-up of 1 year. Therefore, we were unable to ascertain whether the associations differed based on the timing of the occurrence. For instance, a prior study indicated that the association between QoL and MACE could be dependent on the timing [23]. Thirdly, the number of cases in our analyses on MACE were limited. To maintain statistical power, the median was used as the AFEQT questionnaire cut-off instead of quartiles. As such, the distinction between different levels of QoL are less defined in these analyses. Fourthly, statistical floor effects may have influenced the results within this study as patients with EHRA class I at baseline were unable to further improve on their symptoms. As a result, AFEQT quartiles with worse EHRA scores at baseline may have more often been able to improve on their symptoms, likely leading to an overestimation of the strength of association (Supplementary materials S1.4). Lastly, a listwise deletion approach was employed during analysis. As a result, the reported analyses for the various outcomes under study (i.e. MACE, EHRA improvement and hospitalizations) feature separate subsamples, which limits the comparability of results between outcomes. In addition, this strategy may potentially bias results under study if information on confounders was not missing at random.In conclusion, AF-patients with a lower AF-specific QoL at diagnosis were more likely to develop MACE and improve on EHRA score, when compared to patients with a higher QoL at diagnosis. In addition, QoL at diagnosis was also associated with hospitalizations, which was used as a proxy for healthcare costs in this study. As such, this study highlights that the integration of PROs, such as QoL, can be used as a prognostic factor for the expected disease course for AF in daily clinical practice. The routine implementation of PROs will enable care providers to treat more than symptoms and steer on factors that are most relevant to the patient. Therefore, by combining PROs with clinical characteristics of the patient, healthcare professionals are able to provide more patient-centered care, reduce healthcare costs and, as a result, optimize patient value in routine care.

## Electronic supplementary material

Below is the link to the electronic supplementary material.

Supplementary Material 1

## Data Availability

The datasets generated during and/or analysed during the current study are available from the corresponding author on reasonable request.

## Abbreviations

AF: Atrial Fibrillation
MI: Myocardial infarction
MACE: Major Adverse Cardiovascular Events
EHRA score: European Heart Rhythm Association score of atrial fibrillation
QoL: Quality of Life
VBHC: Value-Based Healthcare
AFEQT questionnaire: Atrial Fibrillation Effect on QualiTy-of-life questionnaire
NHN: Netherlands Heart Network
GP: General practitioner
T0: Baseline
T1: 12 months follow-up
MEC-U: Medical research Ethics Committee United
CHA_2_DS_2_-VASc: Congestive heart failure or left ventricular dysfunction Hypertension, Age ≥ 75 (doubled), Diabetes, Stroke, (doubled)-Vascular disease, Age 65–74, Sex category (female)
HAS-BLED: Hypertension, Abnormal renal/liver function, Stroke, Bleeding history or predisposition, Labile INR, Elderly, Drugs/alcohol concomitantly
INR: International Normalized Ratio
BMI: Body Mass Index
DM: Diabetes Mellitus
OSAS: Obstructive sleep apnea syndrome
IBM SPSS: International Business Machines corporation Statistical Package for the Social Sciences
ESC: European Society of Cardiology
PROs: Patient-reported outcomes
PROMs: Patient-reported outcome measures
PCI: Percutaneous coronary intervention
