# Supplementary material for: The prognostic value of quality of life in atrial fibrillation on patient value
Source: Health Qual Life Outcomes. 2023 Apr 5;21:33. doi: 10.1186/s12955-023-02112-2 (PMC10074786; doi:10.1186/s12955-023-02112-2)
Supplement: Supplementary file 1 — Supplementary Material 1 [file 12955_2023_2112_MOESM1_ESM.docx]

**Table S1.1.** Sensitivity analysis between QoL at baseline (AFEQT) and the occurrence of MACE after 12 months of follow-up (T1) taking into account the complete confounder subset.

|  | Total study population |  | MACE (T1-T0) | | | |
| --- | --- | --- | --- | --- | --- | --- |
| AFEQT score (T0) | n (%) |  | n (%)^1^ | OR_minimally-adjusted_ (95% CI) | OR_mv-adjusted_ (95% CI) | *p-value* |
| Below median (4.63 to ≤ 75.93) | 289 (50.6%) |  | 25 (8.7%) | 2.77 (1.29-5.93) | 2.60 (1.18-5.70) | 0.017 |
| Above median (>75.93 to 100) | 282 (49.4%) |  | 10 (3.5%) | 1 (ref.) | 1 (ref.) |  |

Minimally-adjusted models were adjusted for categorized age (<65; ≥65) and gender.

Multivariable-adjusted models were additionally adjusted for HAS_BLED (0-1; ≥2), CHA_2_DS_2_-VASc (0-1; ≥2), type of AF (paroxysmal/persistent), OSAS (yes/no), malignancy (yes/no), heart failure (yes/no), diabetes mellitus (yes/no), BMI (<25 kg/m^2^ ; ≥25kg/m^2^), hypertension (yes/no).

**Table S1.2.** Sensitivity analysis between QoL at baseline (AFEQT) and the improvement in symptom scores (EHRA improvement) after 12 months (T1) taking into account the complete confounder subset..

|  | Total study population |  | EHRA improvement (T1-T0) | | | |
| --- | --- | --- | --- | --- | --- | --- |
| AFEQT score (T0)^e^ | n (%) |  | n (%) | OR_minimally-adjusted_ (95% CI) | OR_mv-adjusted_ (95% CI) | *p-value* |
| First quartile (4.63 to ≤ 57.41) | 110 (30.1%) |  | 66 (40.7%) | 7.58 (3.63-15.83) | 7.36 (3.46-15.68) | <0.001 |
| Second quartile (>57.41 to ≤75.93) | 83 (22.7%) |  | 41 (49.4%) | 4.88 (2.27-10.48) | 4.44 (2.02-9.75) | <0.001 |
| Third quartile (>75.93 to ≤90.74) | 96 (26.3%) |  | 43 (44.8%) | 4.19 (2.00-8.81) | 4.05 (1.89-8.64) | <0.001 |
| Fourth quartile (>90.74 to 100) | 76 (20.8%) |  | 12 (7.4%) | 1 (ref.) | 1 (ref.) |  |

Minimally-adjusted models were adjusted for categorized age (<65; ≥65) and gender.

Multivariable-adjusted models were additionally adjusted for HAS_BLED (0-1; ≥2), CHA_2_DS_2_-VASc (0-1; ≥2), type of AF (paroxysmal/persistent), OSAS (yes/no), malignancy (yes/no), heart failure (yes/no), diabetes mellitus (yes/no), BMI (<25 kg/m^2^ ; ≥25kg/m^2^), hypertension (yes/no).

**Table S1.3.** Sensitivity analysis between QoL at baseline (AFEQT) and AF-related hospitalizations during 12 months of follow-up (T1) taking into account the complete confounder subset..

|  | Total study population |  | Hospitalizations (T1-T0) | | | |
| --- | --- | --- | --- | --- | --- | --- |
| AFEQT score (T0) | n (%) |  | n (%) | OR_minimally-adjusted_ (95% CI) | OR_mv-adjusted_ (95% CI) | *p-value* |
| First quartile (4.63 to ≤ 57.41) | 126 (29.1%) |  | 77 (61.1%) | 3.39 (1.92-5.97) | 3.65 (1.99-6.67) | <0.001 |
| Second quartile (>57.41 to ≤75.93) | 99 (22.9%) |  | 37 (37.4%) | 1.45 (0.71-2.35) | 1.60 (0.85-3.00) | 0.147 |
| Third quartile (>75.93 to ≤90.74) | 110 (25.4%) |  | 36 (32.7%) | 1.07 (0.60-1.93) | 1.20 (0.65-2.22) | 0.562 |
| Fourth quartile (>90.74 to 100) | 98 (22.6%) |  | 30 (30.6%) | 1 (ref.) | 1 (ref.) |  |

Minimally-adjusted models were adjusted for categorized age (<65; ≥65) and gender.

Multivariable-adjusted models were additionally adjusted for HAS_BLED (0-1; ≥2), CHA_2_DS_2_-VASc (0-1; ≥2), type of AF (paroxysmal/persistent), OSAS (yes/no), malignancy (yes/no), heart failure (yes/no), diabetes mellitus (yes/no), BMI (<25 kg/m^2^ ; ≥25kg/m^2^), hypertension (yes/no).

**Table S1.4.** Overall associations between QoL at baseline (AFEQT) and the improvement in symptom scores (EHRA improvement) after 12 months (T1) excluding patients with EHRA I at baseline.

|  | Total study population |  | EHRA improvement (T1-T0) | | | |
| --- | --- | --- | --- | --- | --- | --- |
| AFEQT score (T0)^e^ | n (%) |  | n (%) | OR_minimally-adjusted_ (95% CI) | OR_mv-adjusted_ (95% CI) | *p-value* |
| First quartile (4.63 to ≤ 57.41) | 101 (35.7%) |  | 69 (68.3%) | 1.82 (0.79-4.17) | 1.69 (0.72-3.97) | 0.232 |
| Second quartile (>57.41 to ≤75.93) | 74 (26.1%) |  | 50 (67.6%) | 1.61 (0.71-3.63) | 1.58 (0.69-3.67) | 0.281 |
| Third quartile (>75.93 to ≤90.74) | 70 (24.7%) |  | 49 (70.0%) | 1.69 (0.78-3.70) | 1.38 (0.61-3.10) | 0.439 |
| Fourth quartile (>90.74 to 100) | 38 (13.4%) |  | 22 (57.9%) | 1 (ref.) | 1 (ref.) |  |

Minimally-adjusted models were adjusted for categorized age (<65; ≥65) and gender.

Multivariable-adjusted models were additionally adjusted for HAS_BLED (0-1; ≥2), CHA_2_DS_2_VASc (0-1; ≥2), type of AF (paroxysmal/persistent), Diabetes Mellitus.
